# Role of Plant-Derived Active Constituents in Cancer Treatment and Their Mechanisms of Action

**DOI:** 10.3390/cells11081326

**Published:** 2022-04-13

**Authors:** Abdul Waheed Khan, Mariya Farooq, Muhammad Haseeb, Sangdun Choi

**Affiliations:** 1 Department of Molecular Science and Technology, Ajou University, Suwon 16499, Korea; waheedmarwat31@gmail.com (A.W.K.); mariyafarooq03@gmail.com (M.F.); haseeb3389@hotmail.com (M.H.); 2 S&K Therapeutics, Ajou University Campus Plaza 418, 199 Worldcup-ro, Yeongtong-gu, Suwon 16502, Korea

**Keywords:** cancer, incidence, epidemiology, phytochemicals, mechanism, clinical trials

## Abstract

Despite significant technological advancements in conventional therapies, cancer remains one of the main causes of death worldwide. Although substantial progress has been made in the control and treatment of cancer, several limitations still exist, and there is scope for further advancements. Several adverse effects are associated with modern chemotherapy that hinder cancer treatment and lead to other critical disorders. Since ancient times, plant-based medicines have been employed in clinical practice and have yielded good results with few side effects. The modern research system and advanced screening techniques for plants’ bioactive constituents have enabled phytochemical discovery for the prevention and treatment of challenging diseases such as cancer. Phytochemicals such as vincristine, vinblastine, paclitaxel, curcumin, colchicine, and lycopene have shown promising anticancer effects. Discovery of more plant-derived bioactive compounds should be encouraged via the exploitation of advanced and innovative research techniques, to prevent and treat advanced-stage cancers without causing significant adverse effects. This review highlights numerous plant-derived bioactive molecules that have shown potential as anticancer agents and their probable mechanisms of action and provides an overview of in vitro, in vivo and clinical trial studies on anticancer phytochemicals.

## 1. Introduction

**Figure 1:**
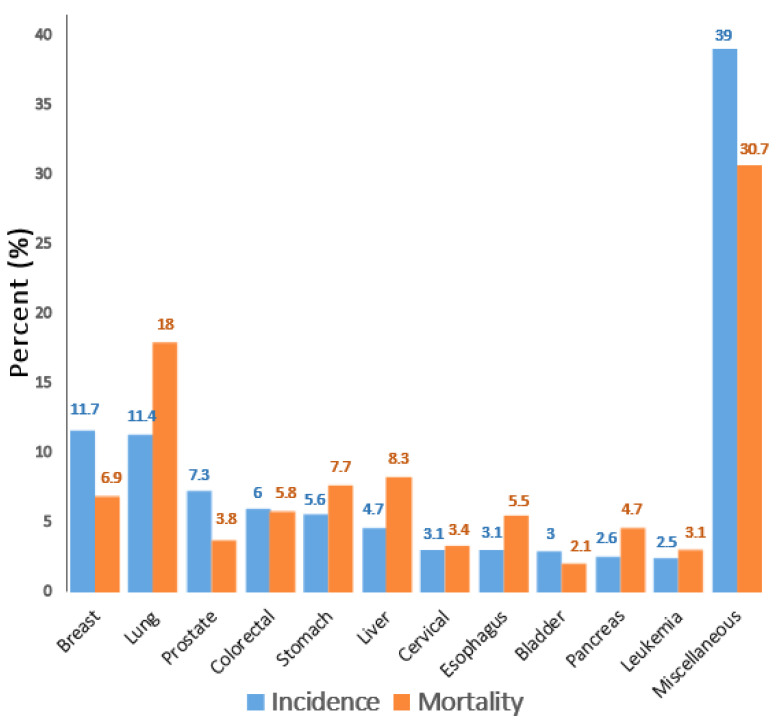
**Incidence and mortality rates of different cancer types in 2020.** Percent increases in incidence and mortality rates of different cancers are shown, with breast, lung, prostate, colorectal, and stomach cancers having the highest incidence and mortality rates. Cancers with low percent incidence and mortality rates are combined as miscellaneous cancers.

Cancer is a challenging disease and is the main cause of mortality worldwide; however, its impact is not evenly distributed. The cancer burden in developed and underdeveloped countries has increased over time owing to a variety of factors, including aging and growing populations, rapid socioeconomic growth, and changes in the incidence of risk factors. Owing to the growth and aging of the world population, cancer is showing reduced survival rates in many countries [1,2]. Cancer is a complex disease involving uncontrolled growth and proliferation of cells in tissues, resulting in cell aggregation locally (tumor), and it can spread to an entire organ or even to other neighboring tissues systemically (metastasis) [3]. The uncontrolled cell behavior can be caused by genetic or epigenetic changes in oncogenes involved in cell proliferation or cell death regulation [4]. The incidence and mortality rates of cancer are continuously increasing. According to a study published in 2020, the global incidence of cancer cases was 247.5, whereas the mortality rate was 127.8 per 100,000 people. Developed countries, such as Japan, Australia, New Zealand, Germany, Canada, and France, topped the list in cancer incidence and mortality rates [2]. Furthermore, breast cancer had the highest incidence rate of 11.7%, while lung cancer had the highest mortality rate of 18% [5]. The worldwide estimated incidence and mortality rates of different cancers are shown in Table 1, and the percentages of incidence and mortality of different types of cancers are shown in Figure 1.

**Figure 2:**
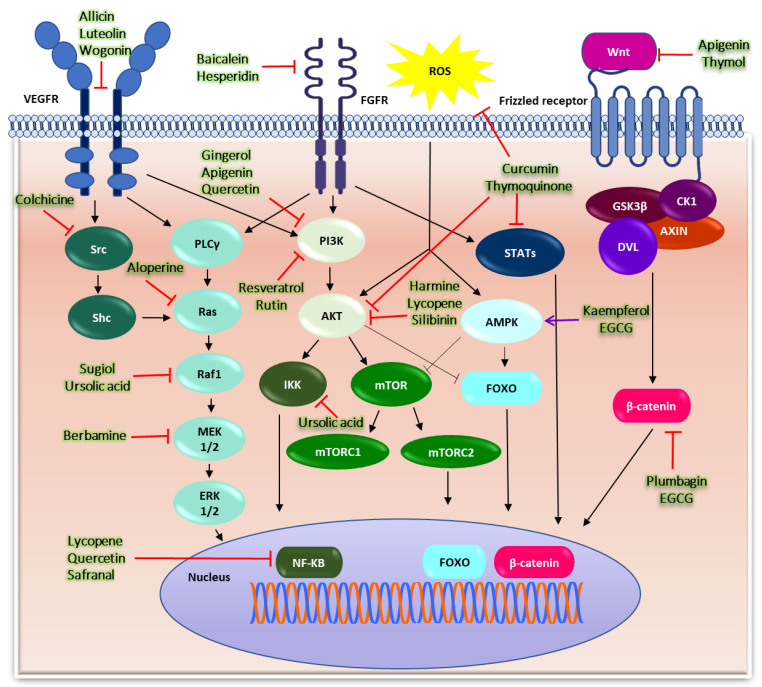
**Important cellular mechanisms involved in cancer and mechanisms of action of phytochemical drugs.** Growth factors, such as vascular endothelial growth factor and fibroblast growth factor, bind with their respective receptors, resulting in their phosphorylation, followed by the activation of downstream signaling pathways, such as the PI3K/Akt, PLCγ, and STAT pathways. Akt activates IKK, which is responsible for the activation of the NF-κB signaling and mTOR pathway; IKK exerts its effect on cells by regulating the hypoxia-induced factor. ROS activates the Akt and AMP-activated protein kinase (AMPK) pathways by inducing endoplasmic reticulum stress. AMPK activates the tumor suppressor transcription factor (FOX O) and inhibits the action of mTOR. Wnt proteins suppress glycogen synthase kinase-3β (GSK-3β) by binding to frizzled receptors, disrupting the β-catenin complex (destructive complex). β-catenin accumulates in the cytoplasm, translocates to the nucleus, and induces cell proliferation, which promotes cancer by activating Wnt-regulated genes. Different phytochemicals act on different targets to exhibit anticancer activity.

Several pathways are involved in cancer development, including the VEGF receptor pathway that can activate the RAS/RAF/MEK/ERK pathway [6] and the fibroblast growth factor (FGF) receptor pathway that activates multiple downward pathways, including the PI3K/Akt/mTOR, RAS/RAF/MEK/ERK and signal transducer and activator of transcription (STAT) pathways [7]. Reactive oxygen species (ROS) can activate the Akt/mTOR and AMPK signaling systems to induce cancer [8]. Wnt/β-catenin also plays a role in the development of multiple cancers [9]. Some important cancer-causing pathways and targets of the anticancer activity of phytochemicals are presented in Figure 2.

Since ancient times, herbal medicines have been used in health care systems. Research conducted to confirm the effectiveness of these medicines led to the discovery and development of plant-based medications. Local communities use medicinal plants to treat most diseases owing to lack of access to modern medication. In the past few decades, increasing evidence has revealed the remarkable potential plant-based therapeutics. Compared with synthetic medicines, medical plants have therapeutic potential with fewer side effects and lower costs [10].

Phytochemicals are plant-derived secondary metabolites. Based on epidemiological, in vitro, in vivo, and clinical trial data, a plant-based diet can lower the risk of many chronic diseases (e.g., neurological diseases, cardiovascular disease, diabetes, and cancer) owing to the action of bioactive plant constituents or phytochemicals [11].

Despite significant progress in the prevention and treatment of cancer, major gaps still exist, and further improvements are warranted. Modern chemotherapy has several side effects that impede the progress of cancer treatment and lead to other serious health problems. The development of integrated research systems and advanced screening procedures for plant bioactive components has ushered in a new era of phytochemical discoveries for the prevention and treatment of complex diseases such as cancer. Bioactive compounds such as berberine, curcumin, crocetin, colchicine, gingerol, lycopene, kaempferol, resveratrol, vincristine, and vinblastine have demonstrated remarkable anticancer potential [4]. Using modern and novel research approaches, more plant-derived constituents might be discovered to prevent and treat advanced-stage cancer without significant side effects. 

In this review, we highlight phytochemicals that have been reported as anticancer agents and their putative mechanisms of action in cancer treatment and summarize in vitro, in vivo, and clinical trial data on these phytoconstituents.

## 2. Methodology

### Data Collection

Articles on phytoconstituents with anticancer activity were searched for using specific keywords such as “phytochemicals”, “plant-derived constituents”, “plant-based medicine”, “antitumor”, “cytotoxic”, “cancer epidemiology,” and “incidence” from online research databases such as PubMed, Web of Science, Medline, Google Scholar, and Science Direct and downloaded. The articles were entirely read, and data on phytochemicals with anticancer properties were collected and tabulated in Table 2. 

## 3. Data Analysis

**Figure 3:**
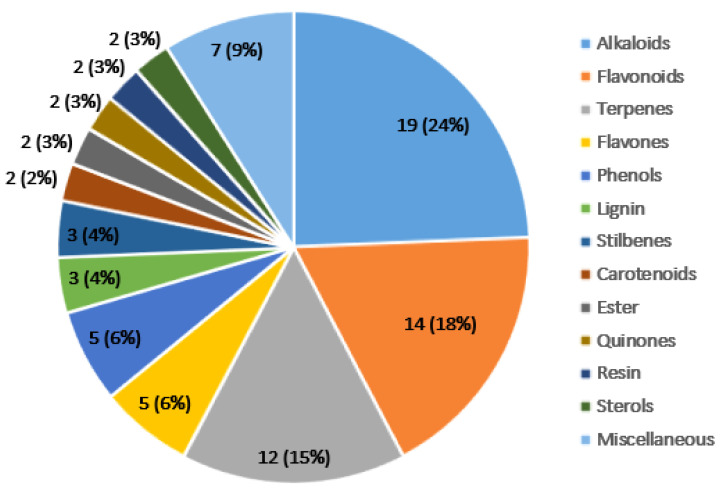
Numbers and percentages of anticancer phytochemicals belonging to different phytochemical classes. In this review, most phytochemicals were found to be constituted of alkaloids followed by flavonoids, terpenes, flavones, and phenols. The phytochemicals classes that have less than two phytochemicals are included in the miscellaneous class.

A total of 78 plant-derived compounds belonging to various families were found to have significant anticancer activity; tested via in vitro and in vivo experiments. Most of these phytochemicals were alkaloids 19 (24%), flavonoids 14 (18%), terpenes 12 (15%), isoflavones 5 (6%), and phenols 5 (6%) (Figure 3).

Multiple phytochemicals were found to exhibit activity against multiple cancers. Most of the phytochemicals were found to be effective against breast (55), lung and colon (53 each), prostate (45), liver (30), ovarian (27), gastric (24), pancreatic (18), cervical (14), bladder (13), skin (11), oral (9), kidney (7), esophageal and thyroid (6 each), bile duct and brain (5 each), and miscellaneous (10) cancers (Table 3).

Of the total phytochemicals, lycopene was found to exhibit activity against 10 different types of cancer; baicalin, corosolic acid, plumbagin, shikonin, and thymoquinone displayed activity against 9; erianin, evodiamine, gallic acid, and gossypol exerted effects against 8; apigenin, curcumin, luteolin, oridonin, resveratrol, and silibinin had effects against 7; and other phytochemicals showed activity against six or less than six types of cancer (Table 4).

**Figure 4:**
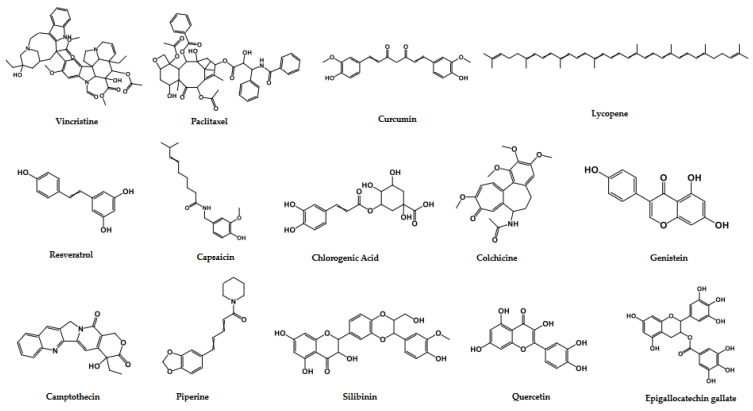
Structures of anticancer phytochemicals approved by FDA or in clinical trials.

Several plant-derived active constituents, such as vincristine, vinblastine, paclitaxel, have been approved by the FDA as therapeutics for different cancers. Several other phytochemicals are currently in clinical trials for the treatment of various cancers (Table 5), and their structures are given (Figure 4).

### 3.1. Important Anticancer Phytochemicals from the Clinical Trials and Their Structure–Activity Relationship Data

According to a scientific report, phytochemicals may have substantial anticancer properties. Approximately 50% of the drugs approved between 1940 and 2014 were obtained directly or indirectly from natural sources [403]. Some important phytochemicals, currently in clinical trials, that showed good in vitro and in vivo potentials in different types of cancers are described below.

### 3.2. Curcumin

Curcumin, a lead phytochemical extracted from *Curcuma longa*, inhibits the growth of human glioma cells by inhibiting numerous cellular and nuclear factors. Curcumin increases the expression of various genes and their products, including p16, p21, and p53, Bax, EIK-1, Erk, c-Jun N-terminal kinase, early growth response protein 1, and caspases-3, -8, and -9, while reducing the expression of Bcl-2, pRB, cyclin D1, mTOR, NF-κB, and p65 [404].

The potent antioxidant property of curcumin is responsible for many of its medicinal actions, including its anticancer activity. The majority of natural antioxidative chemicals are either phenolic or -diketone compounds. But curcumin, is one of the few antioxidative compounds that has both phenolic hydroxy and -diketone groups in a single molecule [405].

In one study, researchers investigated the importance of the phenolic hydroxy groups, and other substituents in the phenyl rings of curcumin and its analogs, to their antioxidant activities by using the three antioxidant bioassays (free radical scavenging activity by the ABTS method, free radical scavenging activity by the DPPH method, and inhibition of lipid peroxidation). In all the three assays, the phenolic curcumin analogs were more potent than the non-phenolic analogs, indicating that the phenolic groups are critical for antioxidant action. Curcumin is thought to be a classic phenolic chain-breaking antioxidant, donating H atoms from phenolic groups [406,407].

In another research study, curcumin analogs were synthesized or isolated from natural sources and evaluated for AR inhibitory activity in prostate cancer cell lines. Among these analogs, few exhibited the greatest inhibitory activity against the transcription of AR, while others showed less or no activity. Based on the bioassay results, researchers showed the SAR of curcumin analogs as anti-AR reagents as follows. (1) The conjugated β-diketone moiety is required for the activity. Saturating or removing the C=C bonds resulted in a decrease or loss of activity, while converting the β-diketone moiety to pyrazole leads to a reduction or loss of activity. (2) When the methylene group in the linker was not substituted, the inhibitory activity was significantly increased by substituting the phenolic hydroxy groups with methoxy or methoxycarbonylmethoxy groups. (3) Adding an ethoxycarbonylethyl group to the central methylene group dramatically improved the anti-AR action of curcumin when the phenyl ring substitution was retained. (4) Anti-AR activity was lost in all electron-withdrawing substitutions in the phenyl rings. The exact mechanism through which curcumin analogs block AR transcription is undisclosed [408,409,410,411]. Further initiatives need to be taken to extend the SAR and enhance anti-AR activities of curcumin.

### 3.3. Epigallocatechin Gallate (EGCG)

EGCG is the chief constituent of green tea that can restore the expression of tumor suppressor genes such as retinoid X receptor-alpha in breast cancer, ultimately preventing breast cancer by binding to other high-affinity proteins such as Zap-70 [412]. EGCG is also found to be effective against lung, colon, and prostate cancers by inducing DNA damage and AMPK signaling and inhibiting Notch1, MMP-2/9, and β-catenin expression [115,117,331].

In EGCG structure, the three aromatic rings are connected by a pyran ring. The structure of EGCG is thought to be responsible for its health-promoting properties. The potent antioxidant effect of catechins is achieved through quinone and semiquinone synthesis, which involves oxidation of phenolic groups with atomic or single electron transfer in the periphery aromatic rings [413,414]. These rings have been linked to a decrease in proteasome activity. Protected analogues are the only ones that suppress proteasome activity. In vitro, dehydroxylation of either one or both periphery aromatic rings, inhibits proteasome inhibitory activity. Furthermore, the apoptotic cell death is induced by these protected analogues in tumor cell-specific manure. These findings showed that the periphery aromatic rings peracetate protected EGCG analogues, have a lot of potential as anti-cancer and cancer-prevention drugs [415]. The first structure–activity correlations between EGCG and heat-shock protein 90 were described and analyzed by Khandelwal et al. His findings suggest that phenolic groups on the aromatic ring, adjacent to pyrin ring, are useful in inhibiting heat-shock protein 90, whereas phenolic substituents on the faraway periphery ring are unfavorable [416]. Finally, when compared to catechins without the 5′-hydroxyl group, the hydroxyl group at the 5′-position in the upper aromatic ring inhibited urease up to 100-fold and also prevented Helicobacter pylori growth in the gut [417].

### 3.4. Genistein

Genistein, a potent anticancer compound, can be isolated from soybeans, lentils, chickpeas, and beans. It exhibits a pro-apoptotic effect in colon cancer and has a variety of functions: it upregulates Bax and p21, blocks topoisomerase II and NF-κB, and increases the expression of antioxidant enzymes such as glutathione peroxidase [418].

Genistein is a natural flavonoid that has been found to interact with several biological targets. After orally administration, its quick breakdown into inactive metabolites and rapid excretion from the body, are the main disadvantages of using genistein as a chemotherapeutic agent [419]. Therefore, to obtain better bioavailability compounds than genistein, a delayed compound metabolism is required. In one study, it was found that the proportion of metabolites was affected by the nature of the glycosidic bond. The metabolization of genistein derivatives with a more stable C-glycosidic bond was slower than derivatives with an O-glycosidic bond. It was also reported that linking a sugar moiety to the genistein structure increases its metabolism time in the body [420].

In another research work, it has been found that in comparison to the genistein parent molecule, novel genistein glycosyl derivatives with an O-glycosidic or C-glycosidic linkage have better antiproliferative effects. [421,422]. The C-7 or C-4′-hydroxyalkyl ethers of genistein (intermediates in the glycoconjugates synthesis), are found to be more active in preventing tumor cell growth than genistein. Furthermore, biological investigations have also revealed that derivatives with a substituent at the C-7 position inhibit the cell cycle in the G2 phase, whereas derivatives with a substituent at the C-4′ position disrupt the cell cycle in the G1 phase. [421]. It is concluded that the structural modification (hydroxyl group etherification) of genistein, successfully improved its antiproliferative activity.

### 3.5. Lycopene

Lycopene is a vibrant red pigment found in tomatoes, red carrots, watermelons, and red papaya. It plays a key role in targeting the PI3K/Akt pathway in stomach and pancreatic cancers by suppressing the expression of Bcl-2, an Erk protein. In breast, endometrial, prostate, and colon cancers, lycopene upregulates antioxidant enzymes GSH, GPxn, and GST and eliminates oxidative injury induced by toxins. Lycopene has been demonstrated to affect the growth and progression of HT-29 cells in culture and tumors in animal models by interfering with numerous cellular signal transduction pathways such as those of JNK and NF-κB. Lycopene also prevents infiltration, metastasis, and multiplication of human SW480 colon cancer cells by inhibiting JNK and NF-κB activation, and suppressing the production of COX-2, IL-1, IL-6, IL-10, and iNOS [423,424].

Carotenoids promoted the expression of phase II enzymes by activating the electrophile/antioxidant response element (EpRE/ARE) transcription pathway. Phase II detoxifying enzymes are a key biological method for minimizing cancer risk. By disrupting the inhibitory effect of Keap1 on Nrf2, the key EpRE/ARE activating transcription factor; certain electrophilic phytonutrients have been demonstrated to stimulate the EpRE/ARE system. However, carotenoids like lycopene are hydrophobic, lacking an electrophilic group, which is unlikely to activate Nrf2 and the EpRE/ARE system directly. The active mediators in lycopene’s activation of the EpRE/ARE system are carotenoid oxidation products. Researchers discovered the main structure–activity rules for EpRE/ARE activation using a series of described mono- and di-apocarotenoids that might potentially be produced from in vivo metabolism of carotenoids (lycopene). Such as active molecules are the aldehydes, not acids; the methyl group on the terminal aldehyde, which regulates the reactivity of the conjugated double bond, is responsible for the activity, and the main chain of the molecule is constituted of the dialdehyde’s optimum length (12 carbons). The apocarotenals suppressed breast and prostate cancer cell proliferation with an efficacy comparable to that of EpRE/ARE activation. These findings may provide a molecular explanation for the cancer-preventive properties of carotenoids like lycopene [425,426].

### 3.6. Resveratrol

Resveratrol, a naturally occurring polyphenol, is found in peanuts, mulberries, grapes, blueberries, and bilberries. It plays a significant role in the treatment of different types of cancers, including colorectal, breast, pancreatic, liver, lung, and prostate cancers, by increasing the expression of Bax and p53 and decreasing the expression of NF-κB, AP-1, Bcl-2, MMPs, cyclins, COX-2, cyclin-dependent kinases, and cytokines. Resveratrol has been recognized to impede angiogenesis and suppress VEGF by decreasing MAP kinase phosphorylation [418].

A research study was carried out to find the structure–activity relationship of resveratrol in cancer. It was observed that the number and position of free phenolic hydroxyl groups have a key role in the anticancer activities of resveratrol. For this purpose, the researchers used different analogs of resveratrol having different phenolic hydroxyl groups for their anticancer activities in T24 cells. They found that the oxyresveratrol (3-OH glycosylated RV, having an extra -OH group than RV) has greater inhibitory effect that RV but polydatin (3-OH glycosylated RV, lack of one -OH group) has a lesser effect than RV. This showed that the increased number of phenolic hydroxyl groups are responsible for the anticancer activity of RV [427]. Herath et al. proved the theory by discovering that when the hydroxyl groups in RV were replaced, the drug’s pharmacological activity decreased [428]. Furthermore, Miksits et al. found that all of RV’s sulfated metabolites were less effective against various cancer cell lines [309]. This suggests that the anti-tumor efficacy of RV can be affected by the conjugation of phenolic hydroxyl groups with sulfuric acid. Hence, again it is proved that the free phenolic hydroxyl groups are important for antitumor effect of RV.

Currently, several investigations on plant-based drugs to treat cancer are ongoing. Some well-known and effective phytochemicals, such as vincristine, were approved by the FDA in 1963 to treat acute leukemia (brand name, Oncovin). Furthermore, paclitaxel was approved for the treatment of metastatic breast cancer, advanced lung cancer, and pancreatic cancer in 2005, 2012, and 2013, respectively, under the brand name, Abraxane. Curcumin, lycopene, and capsaicin, which are under phase-III trials for prostate and breast cancers, are promising candidates for cancer therapy. Quercetin, genistein, silibinin, and EGCG are undergoing clinical trials or treatment for various types of cancers.

This study of anticancer plant-derived phytochemicals will help ethnomedicine and ethnopharmacology investigations, resulting in better outcomes for the medical potential of natural resources. Various phytochemicals highlighted in this review could be further investigated in clinical trials, enabling the availability of more effective anticancer medicines with fewer adverse effects. This study will be beneficial to researchers working on or interested in the discovery of plant-based medicines for treatment of various cancers.

## 4. Conclusions

Researchers have found multiple synthetic drugs for the treatment of cancer, but anticancer drugs are costly and have some major adverse effects like anemia, vital organs damage, and hair and nail loss. Keeping in mind these drawbacks, we searched multiple papers on natural anticancer compounds, their mechanisms, clinicals trials and SAR data of important phytochemicals. The epidemiology data showed that the breast and lung cancers have the highest mortality and prevalence rates. In this study, we found that majority of anticancer compounds belong to alkaloids and flavonoids classes, and the highest number of phytochemicals were found to be effective against breast and lung cancers, which give us a chance to try these phytochemicals in clinical trials and discover some plant-based drugs that control these high spreading cancers. To discover effective anticancer treatments with less side effects and less cost, the world must rely upon, and conduct more research on natural resources, especially plants and their active constituents.

## Figures and Tables

**Table 1:** Estimated worldwide incidence and mortality rates (per 100,000 people) of all cancer types in 2020.

Continents	Incidence	Rank	Mortality	Rank
** Worldwide **	247.5	–	127.8	–
** Asia **	204.8	–	125.2	–
Japan	813.3	1	332.2	3
China	315.6	57	207.5	42
India	96	121	61.5	122
South Korea	449.2	42	172.8	56
** Europe **	587.4	–	261.1	–
Germany	750.2	4	300.9	10
France	716.9	9	284.4	17
Italy	686.8	13	289.0	15
**North America**	693.2	–	189.6	–
USA	689.3	12	185.0	54
Canada	726.9	7	229.7	33
**South America**	224.8	–	109.1	–
Brazil	278.6	63	122.3	72
Argentina	289.6	60	155.0	63
Colombia	222.5	75	108.1	81
**Africa**	82.7	–	53.1	–
South Africa	182.4	83	95.8	87
Morocco	160.8	93	95.5	88
Ethiopia	67.3	158	45.1	155
**Australia**	784.4	2	189.2	51
New Zealand	745.2	5	217.9	38

**Table 2:** Plant-derived phytochemicals with potential anticancer properties, and their mechanisms of action.

Sr #	Phytochemicals	Chemical Nature	Plant’s Source/Origin	Chemical Structure	M: Weight (g/mol)	Cancer Type	Study Type	Targets and Mechanisms
1	Allicin	Thioester	*Allium sativum*	C_6_H_10O_S_2_	162.3	Lung cancer	In vitro	Downregulation of VEGF expression [12]
						Gastric cancer	In vitro	Enhanced expression of p38 and cleavage caspase-3 [13]
						Oral cancer	In vitro	Upregulation of and cleaved caspase-3 [14]
						Brain cancer	In vitro	Elevation in Fas/FasL expression [15]
2	Aloperine	Alkaloid	*Sophora* *alopecuroides*	C_15_H_24_N_2_	232.36	Ovarian cancer	In vitro	Reactive oxygen species activation [16]
						Thyroid cancer	In vitro	Suppression of Akt pathway and downstream B-cell lymphoma (Bcl-2) expression [17]
						Prostate cancer	In vitro, in vivo	Inhibition of Akt and ERK phosphorylation [18]
						Bladder cancer	In vitro	Downregulation of Ras, p-Raf1 and p-Erk1/2 expression [19]
						Colon cancer	In vitro	Inhibition of JAK/Stat3 and PI3K/Akt pathways [20]
						Bones cancer	In vitro	Suppression of PI3K/AKT signaling [21]
3	Alpinumisoflavone	Isoflavone	*Derris eriocarpa*	C_20_H_16_O_5_	336.3	Colon cancer	In vitro	Blockage of DNA repairing [22]
						Esophageal cancer	In vitro, in vivo, ex-vivo	Upregulation of miR-370 and suppression of PIM1 signaling [23]
						Brain cancer	In vitro	Suppression of glycolysis and cyclin D1 expression and activation of caspase-9 [24]
4	Amygdalin	Diglucoside	*Rosaceae kernels*	C_20_H_27_NO_11_	457.4	Bladder cancer	In vitro	Modulation of β1 or β4 integrin expression [25]
						Breast cancer	In vitro	Downregulation of Bcl-2, upregulation of Bax and p38 MAPK signaling pathways [26]
						Prostate cancer	In vitro	Activation of caspase-3 through downregulation of Bcl-2 and up-regulation of Bax [27]
						Cervical cancer	In vitro	Downregulation of Bcl-2 and upregulation of Bax protein [28]
5	Andrographolide	Diterpenoid	*Andrographis* *paniculata*	C_20_H_30_O_5_	350.4	Colon cancer	In vitro	Increase intracellular ROS level [29]
						Skin cancer	In vitro	Activation of JNK and p38 signaling pathway [30]
						Breast cancer	In vitro, in vivo	Suppressing of COX-2 and VEGF pathway [31]
						Prostate cancer	In vitro, in vivo	Facilitate DNA damage [32]
						Bile duct cancer	In vitro	Suppression of Claudin-1 via p-38 pathway [33]
						Ovarian cancer	In vitro	Upregulation of TIMP1 expression [34]
6	Apigenin	Flavonoid	*Matricaria* *chamomilla*	C_15_H_10_O_5_	270.24	Colon cancer	In vitro, in vivo	Inhibition of the Mcl-1, AKT, and ERK pro-survival regulators [35]
						Lung cancer	In vitro, in vivo	Inhibition of NF-κB, AKT and ERK pathway [36]
						Liver cancer	In vitro, in vivo	Inhibition of PI3K/Akt/mTOR signaling [37]
						Pancreatic cancer	In vitro	Through G2/M cell cycle arrest [38]
						Breast cancer	In vitro	Inhibition of YAP/TAZ activity [39]
						Prostate cancer	In vitro, in vivo	Suppression of NF-κB/p65 expression [40]
						Bone cancer	In vitro	Suppression of Wnt/β-catenin signaling [41]
7	Artemisinin	Alkaloid	*Artemisia annua*	C_15_H_22_O_5_	282.33	Colon cancer	In vitro and in vivo	Increase in ROS production [42]
						Kidney cancer	In vitro, in vivo	Inhibition of AKT signaling [43]
						Ovarian cancer	In vitro, in vivo	Suppression of AKT/ERK/mTOR pathway [44]
						Gallbladder cancer	In vitro, in vivo	Inhibition of ERK1/2 pathway [45]
8	Baicalein	Flavonoid	*Scutellaria* *baicalensis*	C_15_H_10_O_5_	270.24	Lung cancer	In vitro, in vivo	Suppression of VEGF, FGFR-2, and RB-1 pathways [46]
						Colon cancer	In vitro	Activation of caspase-3 [47]
						Bladder cancer	In vitro, in vivo	Inhibition of cyclin B1, MMP-2 and MMP-9 mRNA expressions [48]
						Pancreatic cancer	In vitro, in vivo	Increase caspase-3 and Bax, while decrease survivin and Bcl-2 expressions [49]
						Liver cancer	In vitro	Suppression of PI3K/Akt pathway [50]
						Prostate cancer	In vitro	Inhibition of caveolin-1/AKT/mTOR pathway [51]
						Breast cancer	In vitro, in vivo	Activation of PAX8-AS1-N activation [52]
						Ovarian cancer	In vitro, in vivo	Inhibition of YAP and RASSF6 expressions [53]
						Skin cancer	In vitro, in vivo	Inhibition of glucose uptake and metabolism of tumor cells [54]
9	Berbamine	Alkaloid	*Berberis amurensis*	C_37_H_40_N_2_O_6_	608.7	Blood cancer	In vitro	Upregulation of caspase-3 and downregulation of MDR-1 gene expression [55]
						Liver cancer	In vitro, in vivo, ex vivo	Inhibition of Ca2+/Calmodulin-dependent protein Kinase II expression [56]
						Ovarian cancer	In vitro, in vivo	Inhibition of Wnt/β-catenin signaling [57]
						Colon cancer	In vitro	Inhibition of MEK/ERK signaling [58]
						Head & neck cancer	In vitro	Inhibition of STAT3 activation [59]
10	Capsaicin	Capsaicinoid	*Capsicum annuum*	C18H27NO_3_	305.4	Breast cancer	In vitro, in vivo	Downregulation of FBI-1-mediated NF-κB pathway [60]
						Lung cancer	In vivo	Downregulation of MMP-2 and -9 levels [61]
						Prostate cancer	In vitro	Increases protein light chain 3-II (autophagy marker) and ROS levels [62]
						Colon cancer	In vitro	Stabilization and activation of p53 [63]
						Esophageal cancer	In vitro	Decrease hexokinase-2 (HK-2) expression [64]
						Skin cancer	In vitro	Downregulation of PI3-K/Akt/Rac1 pathway [65]
11	Cepharanthine	Alkaloid	*Stephania cepharantha*	C_37_H_38_N_2_O_6_	606.7	Colon cancer	In vitro	Upregulation of p21Waf1/Cip1 pathway [66]
						Breast cancer	In vitro	Inhibition of AKT/mTOR signaling [67]
						Ovarian cancer	In vitro	Increases expression of p21Waf1 and decreasing expression of cyclins A and D proteins [68]
						Liver cancer	In vitro	Activation of JNK1/2 signaling and downregulation of Akt pathway [69]
12	Chlorogenic Acid	Ester	*Etlingera elatior*	C_16_H_18_O_9_	354.31	Liver cancer	In vitro, in vivo	Inhibition of DNMT1 expression [70]
						Colon cancer	In vitro	Activation of PARP-1, and caspase-9 [71]
						Breast cancer	In vitro	Upregulation of Bax and downregulation of Bcl-2 expressions [72]
13	Colchicine	Alkaloid	*Colchicum* *automnale*	C_22_H_25_NO_6_	399.4	Gastric cancer	In vitro, in vivo	Induce caspase-3-mediated mitochondrial apoptosis [73]
						Hypopharyngeal cancer	In vitro, in vivo	Inhibition of phosphorylated FAK/SRC complex and paxillin [74]
						Breast cancer	In vitro	Inhibition of MMP-2 expression [75]
						Colon cancer	In vitro	Decrease in AKT phosphorylation [76]
14	Combretastatin A4	Stilbene	*Combretum caffrum*	C_18_H_20_O_5_	316.3	Lung cancer	In vitro, in vivo	Disruption of microtubule assembly [77]
						Bladder cancer	In vitro, in vivo	Activation of caspase-3 and reduction in BubR1 and Bub3 expressions [78]
						Bone cancer	In vitro	Inhibition of NDRG1 [79]
15	Corosolic acid	Tripernoid	*Lagerstroemia* *speciosa*	C_30_H_48_O_4_	472.7	Lung cancer	In vitro, in vivo	Inhibition of VEGFR2 kinase activity [33]
						Colon cancer	In vitro, in vivo	Inhibition of HER2/HER3 receptors’ heterodimerization [80]
						Gastric cancer	In vitro	Activation of AMPK pathway [81]
						Liver cancer	In vitro, in vivo, ex vivo	Inactivation of CDK19/YAP/O-GlcNAcylation pathway [82]
						Prostate cancer	In vitro, in vivo	Activation of IRE-1/JNK, PERK/CHOP and TRIB3 [83]
						Cervical cancer	In vitro	Downregulation of PI3K and Akt signaling [84]
						Kidney cancer	In vitro	Induction of lipid ROS [85]
						Breast cancer	In vitro	Increase in ROS production and decrease in VEGF concentration [86]
						Bladder cancer	In vitro, in vivo	Upregulation of SQSTM1/P62, NBR1, and UBB expression [87]
16	Crocetin	Carotenoid	*Crocus sativus*	C_20_H_24_O_4_	328.4	Prostate cancer	In vitro, in vivo	Induce DNA damage and apoptosis [88]
						Colon cancer	In vitro	Upregulation FAS/FADD death receptor [89]
						Pancreatic cancer	In vitro, in vivo	Upregulation of Bax and downregulation of Bcl-2 protein [90]
						Gastric cancer	In vitro, in vivo	Upregulation of caspase-3, -8 and -9 [91]
17	Cucurbitacin	Triterpene	*Cucumis sativus*	C_32_H_46_O_8_	558.7	Colon cancer	In vitro	Inhibition of Hippo-YAP Signaling Pathway [92]
						Gastric cancer	In vitro, in vivo	Suppression of Akt expression [93]
						Bile duct cancer	In vitro	Downregulation of pRB, cyclin D1 and cyclin E expression [94]
						Breast cancer	In vitro	Inhibition of Stat3 and Akt signaling [95]
18	Curcumin	Curcuminoids	*Curcuma longa*	C_21_H_20_O_6_	368.38	Breast cancer	In vitro	Upregulation of PTEN/Akt signaling pathway [96]
						Gastric cancer	In vitro	Suppression of PI3K/Akt/mTOR signaling pathway [49]
						Oral cancer	In vivo	Suppression of NF-κB, and COX-2 expression [97]
						Prostate cancer	In vitro	Downregulation of NF-κB, and CXCL1 and -2 expressions [98]
						Colon cancer	In vitro	Inhibition of AMPK-induced NF-κB, uPA, and MMP9 activation [99]
						Ovarian cancer	In vitro	JAK/STAT3 pathway inhibition [100]
						Lung cancer	In vitro	Increase in FOXA2 expression [101]
19	Diosgenin	Saponin	*Dioscorea villosa*	C_27_H_42_O_3_	414.6	Breast cancer	In vitro	Downregulation of Skp2 [102]
						Liver cancer	In vitro	Inhibition of Akt and upregulation of p21 and p27 expression [103]
20	D-limonene	Terpene	*Citrus aurantium*	C_10_H_16_	136.23	Colon cancer	In vitro	Inactivation of Akt pathway [104]
						Lung cancer	In vitro	Upregulation of Atg5 [105]
						Prostate cancer	In vitro	Generation of ROS, and activation of caspase-3 and -9 [106]
21	Emodin	Resin	*Rheum palmatum*	C_15_H_10_O_5_	270.24	Breast cancer	In vitro	Activation of AhR-CYP1A1 signaling pathway [107]
						Lung cancer	In vitro	Suppression of HAS2-HA-CD44/RHAMM pathway [108]
						Pancreatic cancer	In vitro, in vivo	Downregulation of NF-κB, VEGF, MMP-2, and -9 [109]
						Colon cancer	In vitro	Suppression of PI3K/AKT signaling [110]
						Prostate cancer	In vitro	Downregulation of VEGF [111]
22	Epigallocatechin gallate (EGCG)	Catechin	*Camellia sinensis*	C_22_H_18_O_11_	458.4	Bile duct cancer	In vitro, in vivo	Suppression of Notch1, MMP-2, and -9 signaling [112]
						Lung cancer	In vitro	Activation of AMPK signaling pathway [113]
						Ovarian cancer	In vitro	Induce DNA damage [114]
						Prostate cancer	In vitro, in vivo	Inhibition of HSP90 function [115]
						Head & neck cancer	In vitro, in vivo	Inhibition of beta-catenin expression [116]
						Colon cancer	In vitro	Induction of ER stress through PERK/p-eIF2α/ATF4 and IRE1α pathways activation [117]
23	Erianin	Bisbenzyl	*Dendrobium* *chrysotoxum*	C_18_H_22_O_5_	318.4	Breast cancer	In vitro	Activation PI3K/Akt pathway [118]
						Lung cancer	In vitro, in vivo	Induction of Ca2+/CaM-dependent ferroptosis [119]
						Liver cancer	In vitro, in vivo	Induction of oxidative stress-mediated mitochondrial apoptosis [73]
						Oral cancer	In vitro	Regulation of MAPK pathway [120]
						Bladder cancer	In vitro, in vivo	Increase in p-JNK level and induce c-Jun and Bcl-2 phosphorylation [121]
						Bone cancer	In vitro, in vivo	Activation of ROS/JNK signaling [122]
						Colon cancer	In vitro	Activation of JNK pathway [123]
						Cervical cancer	In vitro	Regulation of ERK1/2 signaling [124]
24	Evodiamine	Alkaloid	*Evodia rutaecarpa*	C_19_H_17_N_3_O	303.4	Lung cancer	In vitro, in vivo	Elevation of CD8+ T cells and downregulation of MUC1-C/PD-L1 axis [125]
						Thyroid cancer	In vitro	Through M phase cell cycle arrest and apoptosis’s induction [126]
						Prostate cancer	In vitro	Activation of caspase-3 and -9 [127]
						Liver cancer	In vitro	Deactivation of PI3K/AKT pathway [128]
						Bladder cancer	In vitro	Enhance activation of P38 and JNK signaling [129]
						Colon cancer	In vitro, in vivo	Inhibition of acetyl-NF-κB, p65 and MMP-9 expression [130]
						Ovarian cancer	In vitro	Elevation of p27 and p21, and inhibition of Cdc2 expression [131]
						Pancreatic cancer	In vitro	Inhibition of NF-κB, p65, and Bcl-2 expression, while activate Bax and cleaved caspase-3 [132]
25	Flavopiridol	Flavonoids	*Dysoxylum* *binectariferum*	C_21_H_20_ClNO_5_	41.8	Breast cancer	In vitro	Inhibition of cyclin-dependent kinases [133]
						Thyroid cancer	In vitro, in vivo	Reduction in Cyclin-dependent kinases (CDK) and MCL1 levels [134]
						Bile duct cancer	In vitro, in vivo	Suppression of cyclin-dependent kinase pathway [135]
						Head & neck cancer	In vitro, in vivo	Reduction in cyclin D1 expression [136]
						Lung cancer	In vitro	Reduction in E-cadherin level [137]
						Esophageal cancer	In vitro, in vivo	Decrease in c-Myc expression [138]
26	Gallic Acid	Phenolic acid	*Galanthus nivalis*	C_7_H_6_O_5_	170.12	Lung cancer	In vitro, in vivo	Inhibition of PI3K/Akt pathway [139]
						Liver cancer	In vitro	Suppression of Wnt/β-catenin signaling [140]
						Breast cancer	In vitro, in vivo	Increases expression of cleaved caspase-7, -9, and p53, while reduces expression of Bcl-2, and PARP [141]
						Colon cancer	In vitro, in vivo	Inhibition of SRC and EGFR phosphorylation [142]
						Gastric cancer	In vitro	Increases expression of caspase-3, -8, and P53 gene [143]
						Prostate cancer	In vitro	Generation of ROS [144]
						Ovarian cancer	In vitro, in vivo	Inhibition of carbonic anhydrase IX protein [145]
						Pancreatic cancer	In vitro	Downregulation of protein Bcl-,2 while increases in BAX expression [146]
27	Gambogic acid	Resin	*Garcinia hanburyi*	C_38_H_44_O_8_	628.7	Lung cancer	In vitro, in vivo	Downregulation of Bcl-2, and upregulation of Bax expression [147]
						Breast cancer	In vitro, in vivo	Increase the expression of Fas, cleaved caspase-3, -8, -9 and Bax proteins [148]
						Liver cancer	In vitro	Induces apoptosis through caspases 3, -7, -8 and -9 [149]
						Prostate cancer	In vitro	Induction of ROS production [150]
						Colon cancer	In vitro, in vivo	Inhibition of Akt-mTOR signaling [151]
						Gastric cancer	In vitro, in vivo	Downregulation of circ_ASAP2 and CDK7, while upregulation of miR-33a-5p expression [152]
28	Genistein	Isoflavones	*Glycine max*	C_15_H_10_O_5_	270.24	Liver cancer	In vitro	Upregulation of Bax, cleaved caspase-3 and -9 and downregulation of Bcl-2 expression [153]
						Colon cancer	In vitro, in vivo	Suppression of MiR-95, Akt and SGK1 signaling [154]
						Prostate cancer	In vitro, in vivo	Decrease MMP-2 expression [155]
						Lung cancer	In vitro	Downregulation of FoxM1 [156]
29	Gingerol	Phenol	*Zingiber officinale*	C_17_H_26_O_4_	294.4	Breast cancer	In vitro	Induction of p53-dependent intrinsic apoptosis [157]
						Oral cancer	In vitro	Activate caspases and increase Apaf-1 expression [158]
						Cervical cancer		
						Lung cancer	In vitro, in vivo	Reduction in ROS and iron accumulation and suppression of USP14 expression [159]
						Pancreatic cancer	In vitro	Inhibition of PI3K/AKT signaling [160]
30	Ginkgetin	Flavonoid	*Ginkgo biloba*	C_32_H_22_O_10_	566.5	Breast cancer	In vitro	Downregulation of estrogen receptor [161]
						Lung cancer	In vitro, in vivo	Inhibition of p62/SQSTM1 signaling [162]
						Prostate cancer	In vitro, in vivo	Suppression of STAT3 expression [163]
						Bone cancer	In vitro	Inhibition of STAT3 and activation of caspase-3/9 [164]
						Ovarian cancer	In vitro	Induction of apoptosis by activation of caspase-3 [165]
						Kidney cancer	In vitro	Suppression of JAK2-STAT3 pathway [166]
31	Glycyrrhizin	Triterpenes	*Glycyrrhiza glabra*	C_42_H_62_O_16_	822.9	Breast cancer	In vitro, in vivo	Induces ROS-mediated apoptosis [167]
						Gastric cancer	In vitro	Downregulation of PI3K/AKT pathway [168]
						Prostate cancer	In vitro	Induces DNA damage [169]
						Ovarian cancer	In vitro	Upregulation of Fas and FasL expression [170]
32	Gossypol	Phenol	*Gossypium* *hirsutum*	C_30_H_30_O_8_	518.6	Colon cancer	In vitro	Suppression of genes coding expression for CLAUDIN1, FAS, IL2, and IL8 [171]
						Breast cancer	In vitro	Suppression of IKBKE, CCL2 and MAPK1 expression [172]
						Lung cancer	In vitro	Decrease EGFR phosphorylation and AKT/ERK signaling [173]
						Prostate cancer	In vitro	Activation of p53 protein [174]
						Ovarian cancer	In vitro	Cause changes in thiol/redox states of proteins associated with glycolysis and stress responses [175]
						Cervical cancer	In vitro, in vivo	Inhibition of FAK signaling and reversing TGF-β1-induced EMT [176]
						Head & neck cancer	In vivo	Inhibition of Bcl-X_L_ expression [177]
						Skin cancer	In vitro	Induces mitochondria-dependent apoptosis [178]
33	Harmine	Alkaloid	*Peganum* *harmala*	C_13_H_12_N_2_O	212.25	Breast cancer	In vitro, in vivo	Downregulation of TAZ [179]
						Thyroid cancer	In vitro, in vivo	Downregulation of Bcl-2 and upregulation of Bax expression [180]
						Gastric cancer	In vitro	Inhibition of Akt/mTOR/p70S6K signaling [181]
						Pancreatic cancer	In vitro	Suppression of AKT/mTOR pathway [182]
						Ovarian cancer	In vitro	Inhibition of ERK/CREB pathway [183]
						Lung cancer	In vitro	Suppression of AKT phosphorylation and enhances ROS generation [184]
34	Hesperidin	Flavonoid	*Citrus limon*	C_28_H_34_O_15_	610.6	Lung cancer	In vitro	Downregulation of FGF and NF-κB signal transduction pathways [185]
						Gastric cancer	In vitro	Increase in ROS levels and regulation of MAPK signaling [135]
						Liver cancer	In vitro	Downregulation of Bcl-xL and upregulation of Bax, Bak, and tBid proteins [186]
						Skin cancer	In vitro	Induces DNA damage [187]
						Prostate cancer	In vitro	Induces apoptosis triggered by ROS generation [188]
						Breast cancer	In vitro	Inhibition of PD-L1 expression via downregulation of Akt and NF-κB signaling [189]
35	Hispidulin	Flavone	*Salvia involucrate*	C_16_H_12_O_6_	300.26	Lung cancer	In vitro, in vivo	Induces ROS-mediated apoptosis via ER stress pathway [190]
						Liver cancer	In vitro, in vivo	Upregulation of PPARγ signaling [191]
						Kidney cancer	In vitro, in vivo	Activation of ROS/JNK signaling [192]
						Gastric cancer	In vitro	Activate ERK1/2 and NAG-1 signaling [193]
36	Kaempferol	Flavonoid	*Spinacia oleracea*	C_15_H_10_O_6_	286.24	Breast cancer	In vitro	Increase expression of H2AX, caspase-3, and -9 [194]
						Liver cancer	In vitro	Activation of AMPK signaling [195]
						Kidney cancer	In vitro	Downregulation of AKT and FAK pathways [196]
						Cervical cancer	In vitro	Disruption of mitochondrial membrane potential and intracellular free Ca2+ concentration [197]
						Pancreatic cancer	In vitro	Inhibition of TGM2 expression [198]
						Colon cancer	In vitro	Activation of ATM and p53-Bax axis [199]
37	Kurarinone	Flavonoid	*Sophora* *flavescens*	C_26_H_30_O_6_	438.5	Lung cancer	In vitro, in vivo	Suppression of caspase-7 and -12, and AKT pathway [200]
						Gastric cancer	In vitro	Inhibition of STAT3 signaling [201]
						Breast cancer	In vitro	Inhibition of NF-κB activation [202]
38	Lappaconitine	Diterpenoid	*Aconitum* *sinomontanum*	C_32_H_44_N_2_O_8_	584.7	Colon cancer	In vitro	Downregulation of PI3K/AKT/GSK3β signaling [203]
						Lung cancer	In vitro	Downregulation of Cyclin E1 expression [204]
						Liver cancer	In vitro	Upregulation of Bax, P53, and downregulation of Bcl-2 expressions [205]
39	Licochalcone A	Chalcone	*Glycyrrhiza glabra*	C_21_H_22_O_4_	338.4	Breast cancer	In vitro	Inhibition of PI3K/Akt/mTOR pathway [206]
						Bladder cancer	In vitro	Induces ER stress-dependent apoptosis caused by activation of ER-specific caspase-12 [207]
						Lung cancer	In vitro	Induces ERK and p38 activation while suppresses JNK signaling [208]
						Liver cancer	In vitro	Downregulation of MKK4/JNK [209]
40	Liriodenine	Alkaloid	*Enicosanthellum pulchrum*	C_17_H_9_NO_3_	275.26	Breast cancer	In vitro	Upregulation of p53 [210]
						Lung cancer	In vitro	Lockage of cell cycle progression at the G2/M phase [211]
						Ovarian cancer	In vitro	Inhibition of progression of CAOV-3 cell cycle in S phase [212]
41	Luteolin	Flavonoid	*Reseda luteola*	C_15_H_10_O_6_	286.24	Liver cancer	In vitro	Increases caspase-8 and decreases Bcl-2 expression [213]
						Colon cancer	In vitro	Upregulation of Nrf2 expression [214]
						Gastric cancer	In vitro	Inhibition of STAT3 phosphorylation [215]
						Oral cancer	In vitro	Suppression of EMT-induced transcription factors [216]
						Breast cancer	In vitro	Suppression of NF-κB/c-Myc activation and hTERT transcription [217]
						Pancreatic cancer	In vitro	Inhibition of VEGF expression [218]
						Lung cancer	In vitro	Inhibition of FAK-Src signaling [219]
42	Lycopene	Carotenoid	*Solanum* *lycopersicum*	C_40_H_56_	536.9	Breast cancer	In vitro	Inhibition of Akt phosphorylation [220]
						Prostate cancer	In vitro, in vivo	Downregulation of IL1, IL6, IL8, and TNF-α levels [221]
						Colon cancer	In vitro	Suppression of NF-κB and JNK signaling [222]
						Pancreatic cancer	In vitro	Inhibition of ROS-Mediated NF-κB Signaling [223]
						Lung cancer	In vitro, in vivo	Induction of RARβ expression [224]
						Gastric cancer	In vivo	Increase in SOD, and CAT, while decrease in MDA levels [225]
						Cervical cancer	In vitro	Upregulation of Bax, and downregulation of Bcl-2 expression [226]
						Skin cancer	In vivo	Inhibition of PCNA expression [227]
						Brain cancer	In vitro	Activation of caspase-3 pathway [228]
						Ovarian cancer	In vitro, in vivo	Decrease in integrin α5 expression and MAPK activation [229]
43	Lycorine	Alkaloid	*Crinum asiaticum*	C_16_H_17_NO_4_	287.31	Breast cancer	In vitro, in vivo	Inhibition of STAT3 signaling pathway [230]
						Gastric cancer	In vitro, in vivo	Enhances FBXW7-MCL1 axis level [224]
						Prostate cancer	In vitro, in vivo	Inhibition of JAK/STAT signaling [231]
						Lung cancer	In vitro, in vivo	Inhibition of Wnt/β-catenin signaling [232]
						Liver cancer	In vitro	inhibition of ROCK1/cofilin-induced actin dynamics [233]
44	Magnolol	Lignan	*Magnolia officinalis*	C_18_H_18_O_2_	266.3	Lung cancer	In vitro, in vivo	Downregulation of Akt/mTOR pathway [234]
						Gallbladder cancer	In vitro, in vivo	Increase in p53 expression [235]
						Liver cancer	In vitro	Inhibition of ERK-modulated metastatic process [236]
						Prostate cancer	In vitro	Downregulation of MMP-2 and MMP-9 expression [237]
						Esophageal cancer	In vitro	Activation of MAPK pathway [238]
45	Matrine	Alkaloid	*Sophora flavescens*	C_15_H_24_N_2_O	248.36	Prostate cancer	In vitro	Enhances expression of GADD45B, tumor suppresser gene or AKT/GSK3β/β-catenin [239]
						Ovarian cancer	In vitro, in vivo	Suppression of PI3K/AKT/mTOR pathway expression [240]
						Colon cancer	In vitro	Upregulation of Bax, downregulation of Bcl-2, and activation of caspase-3 and -9 [241]
						Liver cancer	In vitro, in vivo	Upregulation of miR-345-5p and downregulation of circ_0027345 and HOXD3 [242]
						Lung cancer	In vitro	Downregulation of C-C chemokine receptor type 7 (CCR7) [243]
46	Myricetin	Flavonoid	*Myrica nagi Thunb*	C_15_H_10_O_8_	318.23	Thyroid cancer	In vitro	DNA damaging and inducing the release of apoptosis-inducing factor (AIF) [244]
						Bladder cancer	In vitro, in vivo	Activation of caspase-3, and inhibition of Akt and MMP-9 expression [245]
						Colon cancer	In vitro	Increases BAX/BCL2 ratio and AIF release [246]
						Prostate cancer	In vitro	Inhibition of PIM1 and disruption of PIM1/CXCR4 interaction [247]
						Breast cancer	In vitro	Enhances intracellular ROS production [248]
						Lung cancer	In vitro	Inhibition of FAK-ERK signaling pathway [249]
47	Nimbolide	Limonoid triterpene	*Azadirachta indica*	C_27_H_30_O_7_	466.5	Pancreatic cancer	In vitro, in vivo	Reduction in PI3K/AKT/mTOR and ERK signaling [250]
						Colon cancer	In vitro, in vivo	Inhibition of Bcl-x, CXCR4, VEGF, and NF-κB [251]
						Bladder cancer	In vitro	Stimulation of p38 MAPK and AKT phosphorylation [252]
48	Noscapine	Alkaloid	*Papaver* *somniferum*	C_22_H_23_NO_7_	413.4	Colon cancer	In vitro	Inhibition of PI3K/AKT/mTOR pathway [253]
						Breast cancer	In vitro	Decreases NF-κB and increases IκBα expression [254]
						Lung cancer	In vitro, in vivo	Upregulation of PARP, Bax, and repression of Bcl2 expression [255]
						Prostate cancer	In vivo	Suppression of microtubule dynamics [256]
49	Oridonin	Diterpenoid	*Rabdosia rubescens*	C_20_H_28_O_6_	364.4	Colon cancer	In vitro, in vivo	Downregulation of GLUT1 and induction of autophagy [257]
						Liver cancer	In vitro, in vivo	Inhibition of Akt pathway [258]
						Ovarian cancer	In vitro	Suppression of mTOR pathway [259]
						Bladder cancer	In vitro, in vivo	Inactivation of ERK and AKT signaling pathways [260]
						Esophageal cancer	In vitro, in vivo	Suppression of AKT signaling [261]
						Breast cancer	In vitro	Decrease in expression of MMPs and regulation of Integrin β1/FAK pathway [262]
						Bone cancer	In vitro, in vivo	Activation of PPAR-γ and inhibition of Nrf2 pathways [263]
50	Oxymatrine	Alkaloid	*Sophora flavescens*	C_15_H_24_N_2_O_2_	264.36	Cervical cancer	In vitro	Suppression of AKT/mTOR [264]
						Breast cancer	In vitro	Suppress the PI3K/Akt [265]
						Pancreatic cancer	*Ion vitro*	Downregulation of Livin and Survivin expression and upregulation of Bax/Bcl-2 ratio [266]
						Prostate cancer	In vitro, in vivo	Increase in expression of p53 and Bax, and decrease in Bcl-2 level [267]
51	Physapubescin B	Steroid	*Physalis pubescens*	C_30_H_42_O_8_	530.6	Ovarian cancer	In vitro	Suppress transcriptional activity of STAT3 [268]
						Kidney cancer	In vitro, in vivo	Decreases expression of HIF-2α and activation of caspase-3 and -8 [269]
52	Pinostrobin	Flavonoid	*Boesenbergia* *rotunda*	C_16_H_14_O_4_	270.28	Cervical cancer	In vitro	Increases expressions of TRAIL, FADD and production of ROS [270]
						Breast cancer	In vitro	Downregulation of FAK and RhoA signaling [271]
						Lung cancer	In vitro	Via promoting apoptosis [272]
						Prostate cancer	In vitro	Decrease in cyclins B expression [273]
53	Piperine	Alkaloid	*Piper nigrum*	C_17_H_19_NO_3_	285.34	Colon cancer	In vitro	Suppression of Wnt/β-catenin pathway [274]
						Lung cancer	In vitro	Induces p53-mediated cell cycle arrest and apoptosis via activation of caspase-3 and -9 cascades [275]
						Breast cancer	In vitro, in vivo	Induction of cell apoptosis and cell cycle blockage [276]
						Prostate cancer	In vitro	Downregulation of cyclin A & D1 [277]
54	Piperlongumine	Alkaloid	*Piper longum*	C_17_H_19_NO_5_	317.34	Lung cancer	In vitro	Inhibition of Akt phosphorylation [278]
						Prostate cancer	In vitro	Induces DNA damage [279]
						Colon cancer	In vitro	Induces DNA damage via increasing ROS production [280]
55	Plumbagin	Alkaloid	*Plumbago zeylinica*	C_11_H_8_O_3_	188.18	Breast cancer	In vitro	Upregulation of p53 and p21 [281]
						Colon cancer	In vitro	Induction of ROS formation [282]
						Liver cancer	In vitro, in vivo	Downregulation of SIVA/mTOR signaling [283]
						Prostate cancer	In vitro, in vivo	Induction of ROS production, and activation of ER stress [284]
						Lung cancer	In vitro	Activation of caspase-9 and ROS production [285]
						Esophageal cancer	In vitro, in vivo	Inhibition of STAT3-PLK1-AKT signaling [286]
						Bone cancer	In vitro	Downregulation of c-Myc expression [287]
						Cervical cancer	In vitro	Downregulation of MMP 2, 9, β-catenin and N-cadherin, while upregulation of E-cadherin signaling [288]
56	Pristimerin	Triterpenoid	*Mortonia greggii*	C_30_H_40_O_4_	464.6	Colon cancer	In vitro	Decreases in AKT expression [289]
						Oral cancer	In vitro	Inhibition of MAPK/Erk1/2 and Akt signaling [290]
						Prostate cancer	In vitro	Inhibition of HIF-1α [291]
						Lung cancer	In vitro	Downregulation of integrin β1 and MMP2 expression [292]
						Pancreatic cancer	In vitro	Inhibition of Akt/NF-κB/mTOR signaling [293]
57	Pterostilbene	Stilbenoid	*Polygonum* *cuspidatum*	C_16_H_16_O_3_	256.3	Ovarian cancer	In vitro	Decreases release of NF-κB p50, and NF-κB p65 [294]
						Lung cancer	In vitro, in vivo	Enhance ROS generation, caspase-3 activity and ER stress [295]
						Breast cancer	In vitro	Inactivate AKT and mTOR signaling pathways [296]
						Colon cancer	In vitro, in vivo	Facilitate DNA repairing mediated through Top1/Tdp1 pathway [297]
58	Puerarin	Isoflavone	*Pueraria radix*	C_21_H_20_O_9_	416.4	Colon cancer	In vitro	Increase Bax expression and caspase-3 activation [298]
						Prostate cancer	In vitro	Inhibition of Keap1/Nrf2/ARE signaling pathways [299]
						Lung cancer	In vitro, in vivo	Inhibition of PI3K/Akt pathway [300]
						Liver cancer	In vitro	Modulation of MAPK signaling pathway [301]
						Brain cancer	In vitro	Suppression of p-Akt and Bcl-2, while enhancement of Bax and cleaved caspase-3 expression [302]
59	Quercetin	Flavonoid	*Allium cepa*	C_15_H_10_O_7_	302.23	Thyroid cancer	In vitro	Upregulation of Pro-NAG-1/GDF15 [303]
						Breast cancer	In vitro	Inactivation of caspase-3 pathway [304]
						Liver cancer	In vitro	Inhibition of PI3K/Akt and ERK pathways [305]
						Prostate cancer	In vitro	Enhances release of tumor suppressor genes i.e., PTEN, p53 and TSC [306]
						Lung cancer	In vitro	Inhibition of NF-κB Signaling [307]
60	Resveratrol	Stilbenoid	*Polygonum* *cuspidatum*	C_14_H_12_O_3_	228.24	Colon cancer	In vitro	Inactivates PI3K/Akt signaling [308]
						Breast cancer	In vitro	Suppression of Integrin αvβ3 expression [309]
						Ovarian cancer	In vitro	Inactivation of STAT3 signaling [310]
						Pancreatic cancer	In vitro	Suppression of NAF-1 expression, induces ROS accumulation, and activation of Nrf2 signaling [311]
						Gastric cancer	In vitro	Upregulation of Bax, cleaved caspase-3 and -8 while suppression of NF-κB activation [312]
						Lung cancer	In vitro, in vivo	Decreases SIRT1-mediated NF-κB activation [313]
						Skin cancer	In vitro, in vivo	Deacetylation of SIRT1-activated NF-κB [314]
61	Rutin	Flavonoid	*Ruta graveolens*	C_27_H_30_O_16_	610.5	Colon cancer	In vitro	Inhibition of caspase-3 expression [315]
						Brain cancer	In vitro	Upregulation of P53 expression [265]
						Skin cancer	In vitro	Suppression of PI3K/Akt and Wnt/β-catenin signaling [316]
						Breast cancer	In vitro, in vivo	Inhibition of tyrosine kinase c-Met receptor [317]
62	Safranal	Alkaloid	*Crocus sativus*	C_10_H_14_O	150.22	Colon cancer	In vitro	Suppression of PI3K/Akt/ mTOR pathway [318]
						Liver cancer	In vitro	Activation of caspases-8 and -9 [319]
						Prostate cancer	In vitro, in vivo	Downregulation of AKT and NF-κB signaling [320]
						Breast cancer	In vitro	Inhibits DNA and RNA synthesis [321]
63	Shikonin	Quinone	*Lithospermum erythrorhizon*	C_16_H_16_O_5_	288.29	Lung cancer	In vitro	Downregulation of PFKFB2 expression [322]
						Colon cancer	In vitro	Reduction in peroxiredoxin V (PrxV) expression [323]
						Prostate cancer	In vitro	Induces necroptosis by decreasing caspase-8 and increasing pRIP1 and pRIP3 [324]
						Liver cancer	In vitro, in vivo	Inhibition of PKM2 expression [325]
						Ovarian cancer	In vitro	Decreases Bcl-2 expression and increases BAX, caspase-3 and -9 expression [326]
						Skin cancer	In vitro, in vivo	Inhibition of MAPK pathway-mediated induction of apoptosis [327]
						Bile duct cancer	In vitro	Inhibitions of PKM2 expression [328]
						Breast cancer	In vitro	Inhibition of epidermal growth factor receptor signaling [329]
64	Shogaol	Phenol	*Zingiber officinale*	C_17_H_24_O_3_	276.4	Breast cancer	In vitro	Inhibition Akt and STAT signaling pathway [330]
						Prostate cancer	In vitro, in vivo	Inhibition of STAT3 and NF-κB signaling [331]
						Lung cancer	In vitro, in vivo	Inhibits secretion of CCL2 [332]
						Cervical cancer	In vitro	Induces apoptosis and G2/M cell cycle arrest [333]
65	Silibinin	Flavonolignan	*Silybum marianum*	C_25_H_22_O_10_	482.4	Breast cancer	In vivo	Inhibition of EGF–EGFR signaling pathway [334]
						Lung cancer	In vitro, in vivo	Activation of EGFR/LOX pathway [335]
						Ovarian cancer	In vitro, in vivo	Inhibition of ERK and Akt pathway [336]
						Prostate cancer	In vitro	Suppression of vimentin and MMP-2 expression [337]
						Skin cancer	In vivo	Via Pro-Oxidant activity [338]
						Colon cancer	In vitro	Downregulation of COX-2, VEGF, MMP-2, & -9, and CXCR-4 expression [339]
						Gastric cancer	In vitro	Inhibition of STAT3 pathway [340]
66	Silymarin	Flavonolignan	*Silybum marianum*	C_25_H_22_O_10_	482.4	Oral cancer	In vitro, in vivo	Induction of DR5/caspase-8 apoptotic signaling [289]
						Gastric cancer	In vitro	Inhibition of p-ERK and activation of p-p38 and p-JNK pathways [341]
						Colon cancer	In vitro	Increases ATF3 transcription through activation of JNK and IκK-α [291]
						Prostate cancer	In vitro	Inhibition of cyclins (A, B1, D, E) and cyclin-dependent kinase pathway [337]
						Breast cancer	In vitro, in vivo	Regulation of MAPK signaling pathway [342]
						Liver cancer	In vivo	Reduction in ROS levels [343]
67	Solamargine	Alkaloid	*Solanum nigrum L.*	C_45_H_73_NO_15_	868.1	Gastric cancer	In vitro, in vivo	Inhibition of Erk1/2 MAPK phosphorylation [344]
						Skin cancer	In vitro	Downregulation of hILP/XIAP [345]
						Bone cancer	In vitro	Suppression of notch pathway [346]
						Liver cancer	In vitro	Induction of apoptosis [347]
						Prostate cancer	In vitro, in vivo	Suppression of MUC1 expression [348]
68	Stachydrine	Alkaloid	*Herba Leonuri*	C_7_H_13_NO_2_	143.18	Breast cancer	In vitro	Inhibition of Akt/ERK pathways [349]
						Prostate cancer	In vitro	Inhibits CXCR3 and CXCR4 expressions [350]
69	Sugiol	Diterpene	*Salvia prionitis*	C_20_H_28_O_2_	300.4	Ovarian cancer	In vitro	Blockage of RAF/MEK/ERK signaling pathway [351]
						Prostate cancer	In vitro, in vivo	Inhibits STAT3 activity and increase ROS level [352]
						Pancreatic cancer	In vitro	Induces ROS-mediated alterations in MMP [353]
						Uterine cancer	In vitro	Increases Bax and decreases Bcl-2 expressions [354]
70	Tanshinone	Terpenoids	*Salvia miltiorrhiza*	C_18_H_12_O_3_	276.3	Lung cancer	In vitro, in vivo	Suppression of IL-8 through NF-κB and AP-1 Pathways [355]
						Gastric cancer	In vitro, in vivo	Downregulation of STAT3 pathway [356]
						Breast cancer	In vitro	Suppression of HIF-1α and VEGF [357]
						Ovarian cancer	In vitro, in vivo	Downregulation of Bcl-2, VEGF, COX2 and upregulation of Bax expressions [358]
						Bladder cancer	In vitro	Activation of caspases 3 and -9 [359]
						Cervical cancer	In vitro	Decrease in Bcl-2, HPV 16 and E7 protein levels, while increase in Bax and caspase-3 expressions [360]
71	Tectochrysin	Flavonoids	*Alpinia oxyphylla*	C_16_H_12_O_4_	268.26	Colon cancer	In vitro	Inhibition of NF-κB signaling [361]
						Prostate cancer	In vitro	Suppression of PI3K/AKT pathway [362]
						Lung cancer	In vitro	Inhibition of STAT3 signaling [363]
72	Tetrandrine	Alkaloid	*Stephania tetrandra*	C_38_H_42_N_2_O_6_	622.7	Cervical cancer	In vitro, in vivo	Downregulation of MMP2 and MMP9 [364]
						Breast cancer	In vivo	Upregulation of Caspase-3, Bax, and downregulation of Bcl-2, Survivin, and PARP signaling [365]
						Gastric cancer	In vitro, in vivo	Activation of caspase-3 and -9, and upregulation of apaf-1 [366]
						Colon cancer	In vitro	Inhibition of EMT transition [367]
						Prostate cancer	In vitro	Induction of DR4 and DR5 expression, and TRAIL-mediated apoptosis [368]
						Bone cancer	In vitro, in vivo	Inhibition of PTEN/Akt, MAPK/Erk and Wnt signaling pathways [369]
73	Thymol	Phenol	*Thymus vulgaris*	C_10_H_14_O	150.22	Lung cancer	In vitro	Enhances cytoplasmic membrane permeability and cell apoptosis [370]
						Breast cancer		
						Prostate cancer		
						Colon cancer	In vitro	Suppression of Wnt/β-Catenin pathway [371]
						Gastric cancer	In vitro	Activation of Bax, PARP, and caspase-8 proteins [372]
74	Thymoquinone	Quinone	*Nigella sativa*	C_10_H_12_O_2_	164.2	Kidney cancer	In vitro	Inhibition of AKT phosphorylation [373]
						Breast cancer	In vitro, in vivo	Through phosphorylation of p38 via ROS generation [374]
						Bladder cancer	In vitro	Inhibition of mTOR signaling [375]
						Colon cancer	In vitro	Inhibition of STAT3, JAK2- and EGF receptor tyrosine kinase [376]
						Gastric cancer	In vitro, in vivo	Inhibition of STAT3 pathway [377]
						Liver cancer	In vitro	Inhibition of IL-8 expression, and activation of TRAIL receptors [378]
						Lung cancer	In vitro	Reduction in ERK1/2 phosphorylation [379]
						Oral cancer	In vitro	Downregulation of p38β MAPK [380]
						Pancreatic cancer	In vitro	Downregulation of mucin 4 expression [381]
75	Ursolic acid	Triterpenoids	*Oldenlandia diffusa*	C_30_H_48_O_3_	456.7	Ovarian cancer	In vitro	Downregulation of PI3K/AKT pathway [382]
						Lung cancer	In vitro	Enhances apoptosis-inducing factor (AIF) and endonuclease G release [383]
						Colon cancer	In vitro, in vivo	Inhibition of IL-6-mediated STAT3 pathway [384]
						Breast cancer	In vitro	Downregulation of Nrf2 expression [385]
						Pancreatic cancer	In vitro, in vivo	Inhibition of NF-κB and STAT3 pathways [386]
						Gallbladder cancer	In vitro	Activation of caspase-3, -9 and PARP pathway [387]
76	Withaferin-A	steroidal lactone	*Withania somnifera*	C_28_H_38_O_6_	470.6	Breast cancer	In vitro	Inhibition of TASK-3 expression [388]
						Oral cancer	In vitro	Upregulation of Bim and Bax expression [389]
						Skin cancer	In vitro	Activation of TRIM16 [390]
						Bone cancer	In vitro	Inactivation of Notch-1 signaling [391]
						Colon cancer	In vitro, in vivo	Inhibition of STAT3 Transcriptional activity [392]
77	Wogonin	Flavonoid	*Scutellaria* *baicalensis*	C_16_H_12_O_5_	284.26	Colon cancer	In vitro	Increases ER stress, and mediates p53 phosphorylation [393]
						Cervical cancer	In vitro	Inhibition of Cdk4 and cyclin D1 [394]
						Lung cancer	In vitro	Downregulation of SGK1 protein levels [395]
						Bone cancer	In vitro	Increases ROS level [396]
						Breast cancer	In vitro	Activation of ERK and p38 MAPKs pathways [397]
						Ovarian cancer	In vitro	Increase in p53 and decrease in VEGF proteins expression [398]
78	Xanthatin	Sesquiterpene lactone	*Xanthium* *strumarium*	C_15_H_18_O_3_	246.3	Skin cancer	In vitro, in vivo	Inhibition of Wnt/β-catenin pathway [399]
						Lung cancer	In vitro, in vivo	Inhibition of GSK-3β signaling [400]
						Breast cancer	In vitro, in vivo	Inhibition of VEGFR2 signaling [401]
						Colon cancer	In vitro	Inhibition of mTOR pathway [402]

**Table 3:** Number of effective phytochemicals against different types of cancer.

Cancer Type	Number of Phytochemicals	Cancer Type	Number of Phytochemicals	Cancer Type	Number of Phytochemicals
Breast cancer	55	Pancreatic cancer	18	Esophageal cancer	6
Colon cancer	53	Cervical cancer	14	Thyroid Cancer	6
Lung cancer	53	Bladder cancer	13	Bile duct cancer	5
Prostate cancer	45	Bladder cancer	13	Brain cancer	5
Liver cancer	30	Skin cancer	11	Miscellaneous	10
Ovarian Cancer	27	Oral cancer	9	NA	NA
Gastric cancer	24	Kidney cancer	7	NA	NA

**Table 4:** Phytochemicals with activity against different number of cancer types.

Sr #	Phytochemicals	Effective against Number of Cancer Types
1	Lycopene	10
2	Baicalin, Corosolic acid, Plumbagin, Shikonin, Thymoquinone	9
3	Erianin, Evodiamine, Gallic acid, Gossypol	8
4	Apigenin, Curcumin, Luteolin, Oridonin, Resveratrol, Silibinin	7
5	Other phytochemicals	≤6

**Table 5:** List of phytochemicals approved by the FDA or in clinical trials for various types of cancer.

Sr #	Phytochemicals	Source	Cancer Type	Development Stage	Status	Trade Name	NCT Number
1	Vincristine	*Catharanthus roseus*	Acute leukemia	FDA approved	1963	Oncovin	NA
2	Paclitaxel	*Taxus braciola*	Late-stage pancreatic cancer	FDA approved	2013	Abraxane^®^	NA
			Advanced non-small cell lung cancer	FDA approved	2012	Abraxane^®^	NA
			Metastatic breast cancer	FDA approved	2005	Abraxane^®^	NA
3	Curcumin	*Curcuma longa*	Prostate cancer	Phase 3	Recruiting, 15 June 2021	Biocurcumax (BCM-95) ^®^	NCT03769766
			Cervical cancer	Phase 2	Not yet recruiting, 25 June 2021	Curcugreen (BCM-95) ^®^	NCT04294836
			Pancreatic cancer	Phase 2	Recruiting, 2020	NA	NCT00094445
			Gastric cancer	Phase 2	Not yet recruiting, 13 January 2022	Meriva^®^	NCT02782949
			Breast cancer	Phase 1	Recruiting, 23 February 2021	NA	NCT03980509
4	Lycopene	*Solanum lycopersicum*	Prostate cancer	Phase 3	Completed, 23 January 2018	NA	NCT01105338
5	Resveratrol	*Polygonum* *cuspidatum*	Multiple myeloma cancer	Phase 2	Terminated (collecting more data) 27 February 2019	SRT501	NCT00920556
			Colon cancer	Phase 1	Completed, 14 June 2017	SRT501	NCT00920803
			Neuroendocrine cancer	NA	Completed, 18 November 2019	NA	NCT01476592
6	Capsaicin	*Capsicum annuum*	Breast cancer	Phase 3	Recruiting, 29 December 2021	Qutenza^®^	NCT03794388
			Head and neck cancer	Phase 2	Recruiting, 5 August 2021	Qutenza^®^	NCT04704453
			Prostate cancer	Phase 2	Not yet recruiting, 16 January 2014	Cayenne	NCT02037464
7	Chlorogenic acid	*Etlingera elatior*	Lung cancer	Phase 2	Recruiting, 26 November 2018	NA	NCT03751592
8	Colchicine	*Colchicum autumnale*	Liver cancer	Phase 2	Recruiting, 11 February 2020	Colchicine	NCT04264260
9	Genistein	*Glycine max*	Prostate cancer	Phase 2	Temporarily suspended, 4 December 2020	NA	NCT02766478
			Colorectal cancer	Phase 2	Completed, 10 May 2019	Bonistein	NCT01985763
			Prostate cancer	Phase 2	Completed, 6 August 2019	Novasoy 400	NCT01036321
			Bladder cancer	Phase 2	Completed, 10 June 2021	NA	NCT00118040
10	Camptothecin	*Camptotheca acuminata*	Solid tumor	Phase 2	Completed, 28 May 2020	CRLX101	NCT00333502
			Stomach and esophageal cancer	Phase 2	Completed, 1 February 2018	CRLX101	NCT01612546
			Advanced non-small cell lung cancer	Phase 2	Completed, 28 May 2020	CRLX101	NCT01380769
11	Piperine	*Piper nigrum*	Prostate cancer	Phase 2	Not yet recruiting, 3 November 2021	NA	NCT04731844
12	Silibinin	*Silybum marianum*	Prostate cancer	Phase 2	Completed, 31 March 2014	Silibin-Phytosome	NCT00487721
13	Quercetin	*Allium cepa*	Squamous cell carcinoma	Phase 2	Recruiting, 28 October 2021	NA	NCT03476330
14	Epigallocatechin gallate	*Camellia sinensis*	Colon cancer	Phase 1	Recruiting, 15 December 2021	Teavigo^™^	NCT02891538
			Esophageal cancer	Phase 1	Recruiting, 10 September 2021	NA	NCT05039983

## Data Availability

Not applicable.

## Abbreviations

AIF	Apoptosis-inducing factor	MUC1-C	Mucin 1, cell surface associated protein
Apaf-1	Apoptotic protease activating factor 1	NAF-1	Nuclear assembly factor 1
ATF4	Activating transcription factor 4	NAG-1	NSAID activated gene 1
Bcl-X_L_	B-cell lymphoma-extra large	NBR1	Neighbor of BRCA1 gene 1
CCL2	Chemokine (C-C motif) ligand 2	Nrf2	Nuclear factor erythroid 2–related factor 2
CDK	Cyclin-dependent kinases	PD-L1	Programmed death-ligand 1
CHOP	C/EBP homologous protein	PKM2	Pyruvate kinase M2
CREB	cAMP-response element binding protein	PLK1	Polo-like kinase 1
CXCR4	C-X-C chemokine receptor type 4	PPARγ	Peroxisome proliferator- activated receptor gamma
DR5	Death receptor 5	PTEN	Phosphatase and tensin homolog deleted in chromosome 10
ER	Endoplasmic reticulum	Raf	Rapidly accelerated aibrosarcoma
FAK	Focal adhesion kinase	RASSF6	Ras-association domain family
FOXA2	Forkhead box protein A2	RHAMM	HMMR hyaluronan-mediated motility receptor
GADD45B	Growth arrest and DNA-damage-inducible, beta protein	RhoA	Ras-homolog family member A
GLUT1	Glucose transporter 1	RIP1	Receptor interacting protein 1
H2AX	H2A histone family member X	ROCK1	Rho-associated protein kinase 1
HIF-2α	Hypoxia inducible factor 2 alpha	ROS	Reactive oxygen species
HMGB1	High mobility group box 1 protein	SGK1	Serum/glucocorticoid regulated kinase 1
HOXD3	Homeobox D3	Skp2	S-phase kinase associated protein 2
HSP90	Heat shock protein 90	TASK-3	Two-pore-domain acid sensitive K^+^ channel 3 TASK-3
hTERT	Human telomerase reverse transcriptase	TGF-β1	Transforming growth factor-beta1
iNOS	Inducible nitric oxide synthase	TNF-α	Tumor necrosis factor alpha
IκBα	IkappaB alpha	Top1	Topoisomerase 1
IκK-α	Inhibitory-κB kinase alpha	TRAIL	TNF-related apoptosis-inducing ligand
JNK	Jun N-terminal kinase	TRIM16	Tripartite motif-containing protein 16
Keap1	Kelch-like ECH-associated protein 1	uPA	Urokinase-type plasminogen activator
LOX	Lysyl oxidase	USP14	Ubiquitin specific peptidase 14
MEK	MAPK/ERK kinase	Wnt	Wingless-related integration site
mTOR	Mammalian target of rapamycin	XIAP	X-linked inhibitor of apoptosis protein
